# Discovery of an OTUD3 inhibitor for the treatment of non-small cell lung cancer

**DOI:** 10.1038/s41419-023-05900-2

**Published:** 2023-06-27

**Authors:** Yonghui Zhang, Tongde Du, Na Liu, Juan Wang, Lingqiang Zhang, Chun-Ping Cui, Chaonan Li, Xin Zhang, Bo Wu, Jinhao Zhang, Wenli Jiang, Yubing Zhang, Yuting Zhang, Hongchang Li, Peiyu Li

**Affiliations:** 1 Medical School of Chinese PLA, Beijing, 100853 China; 2 Senior Department of General Surgery, The First Medical Center, Chinese PLA General Hospital, Beijing, 100853 China; 3 Suzhou Institute of Systems Medicine, Suzhou, Jiangsu 215000 China; 4 Department of Oncology, Dushu Lake Hospital Affiliated to Soochow University, Medical Center of Soochow University, Suzhou Dushu Lake Hospital, Suzhou, Jiangsu 215123 China; 5 State Key Laboratory of Proteomics, National Center for Protein Sciences (Beijing), Beijing Institute of Lifeomics, Beijing, 100850 China; 6 Department of Cell Biology, School of Basic Medical Sciences, Anhui Medical University, Hefei, Anhui 230032 China; 7 Department of Cell Biology, School of Basic Medicine, Medical College, Qingdao University, Qingdao, Shandong 266071 China; 8 School of Life Sciences, Hebei University, Baoding, Hebei 071002 China

**Keywords:** Ubiquitylation, Drug development

## Abstract

The ubiquitin-proteasome system (UPS) controls protein turnover, and its dysfunction contributes to human diseases including cancer. Deubiquitinating enzymes (DUBs) remove ubiquitin from proteins to maintain their stability. Inhibition of DUBs could induce the degradation of selected oncoproteins and has therefore become a potential therapeutic strategy for cancer. The deubiquitylase OTUD3 was reported to promote lung tumorigenesis by stabilizing oncoprotein GRP78, implying that inhibition of OTUD3 may be a therapeutic strategy for lung cancer. Here, we report a small-molecule inhibitor of OTUD3 (named OTUDin3) by computer-aided virtual screening and biological experimental verification. OTUDin3 exhibited pronounced antiproliferative and proapoptotic effects by inhibiting deubiquitinating activity of OTUD3 in non-small-cell lung cancer (NSCLC) cell lines. Moreover, OTUDin3 efficaciously inhibited growth of lung cancer xenografts in mice. In summary, our results support OTUDin3 as a potent inhibitor of OTUD3, the inhibition of which may be a promising therapeutic strategy for NSCLC.

## Introduction

The ubiquitin proteasome system (UPS) is essential for life in eukaryotes and regulates many aspects of cell physiology [1, 2]. Ubiquitin (Ub) is covalently conjugated to protein substrates through the concerted action of activating (E1), conjugating (E2), and ligase (E3) enzymes and is removed by deubiquitinating enzymes (DUBs) [3]. Dysfunction of the ubiquitin proteasome system (UPS) has been implicated in the etiology and progression of a diverse set of human diseases including cancer, metabolic syndromes, neurodegeneration, autoimmunity, inflammatory disorders, and infection [4–9]. The UPS plays fundamental roles in the regulation of protein homeostasis and functions that are often altered during cancer progression, thus providing entry points for the development of anti-tumor therapies. The approval and clinical success of the proteasome inhibitor Velcade (bortezomib) and its successors has validated the UPS as a viable target for therapeutic intervention [10]. As part of the UPS pathway upstream of the proteasome, DUBs may offer new opportunities for the development of novel therapeutics with potentially enhanced specificity and reduced toxicity [11]. DUB inhibition increases ubiquitination of specific substrates without affecting global protein or ubiquitin levels, which is among the most exciting and clinically applicable use of DUB inhibitors [12].

There are ~100 identified human DUBs, which cluster by the sequence similarity of their respective catalytic domain into seven families: ubiquitin-specific proteases (USPs), ovarian tumor proteases (OTUs), ubiquitin C-terminal hydroxylases (UCHs), Machado-Josephin domain proteases (MJDs), Jab1/Mov34/Mpr1 Pad1 N-terminal+ proteases (JAMMs), motif interacting with ubiquitin-containing novel DUB family proteases (MINDYs), and zinc finger containing ubiquitin peptidase 1 (ZUP1) [13]. USPs, as the largest subfamily of DUBs, have attracted the most attention on inhibitors research and development [12]. Several inhibitors have been verified as effective in battling malignant tumors, especially those targeting USP1, USP7 and USP14, which display great potential for clinical application [14–22].

OTUD3, a member of the OTUs, has attracted increasing interest in recent years. The studies have shown that OTUD3 plays a key role in tumorigenesis, neurodegeneration, metabolism and immunity [23–29]. Remarkably, OTUD3 promotes lung tumorigenesis by stabilizing the glucose-regulated protein 78 kDa (GRP78) [26], which is a multifunctional protein with activities far beyond its well-known role in controlling unfolded protein response and implicated in promoting tumor proliferation, metastasis and involved in drug resistance [30–36]. This implies that targeted inhibition of OTUD3 may be a potential strategy for the treatment of lung cancer.

To date, no small-molecule inhibitors of OTUD3 have been reported. In this study, we screened 100 000 compounds available at ChemDiv for small-molecule inhibitors of OTUD3 by structure-based virtual screening. From this, we identified several primary hits that were further validated for inhibition of lung cancer cell growth by proliferation assays. OTUDin3, one of the candidate compounds, was identified using cell proliferation assays in vitro. Then, we confirmed that OTUDin3 bound to OTUD3 and inhibited the deubiquitinating activity of OTUD3 by interfering with Ub binding to OTUD3. Next, we demonstrated that OTUDin3 inhibited non-small-cell lung cancer (NSCLC) cell lines by enhancing the degradation of GRP78 both in vitro and in vivo. Collectively, we identified the first small-molecule inhibitor of OTUD3, which effectively inhibited NSCLC. Our findings support inhibition of OTUD3 as a promising therapeutic strategy to treat NSCLC.

## Results

### Identification of small-molecule OTUD3 inhibitors

Previously, discovery of novel targeted inhibitors for DUBs has been mainly performed using high-throughput screening (HTS) of small molecule libraries [12]. Compared with traditional drug discovery methods, computer-aided drug design (CADD) is more efficient and economical. CADD integrates molecular docking to the ligand-binding pocket of a promising therapeutic target, computes the binding energy of each docked small-molecule compound, and selectively chooses the best ones as candidates for subsequent experimental procedures. Therefore, in this study we employed computational virtual screening based on molecular docking for the identification of OTUD3 inhibitors.

**Fig. 1:**
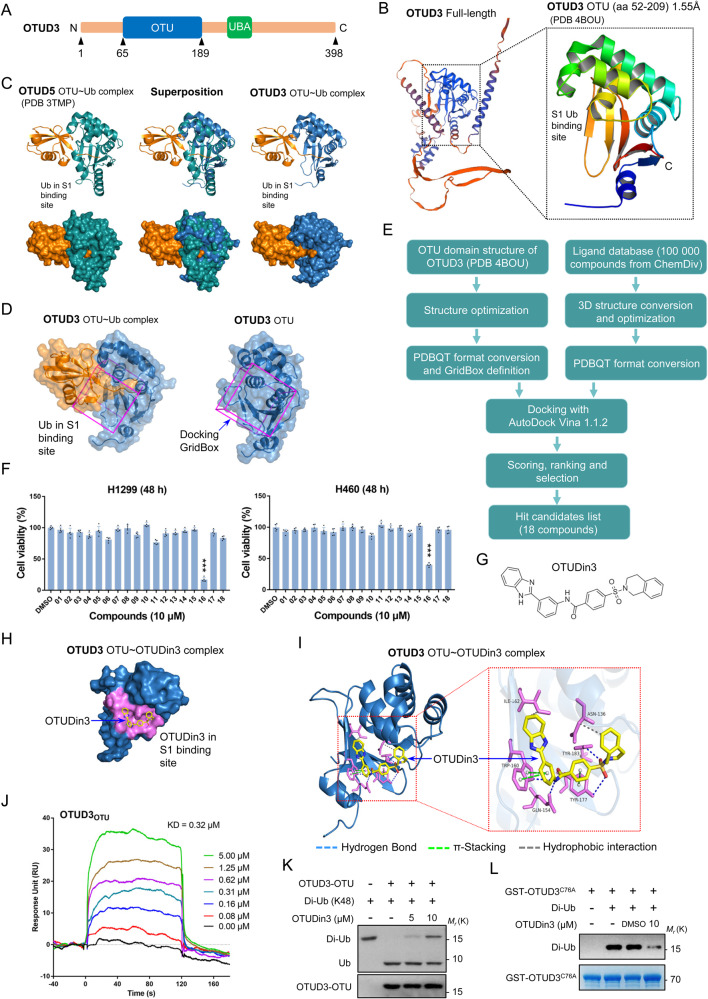
Identification of small-molecule OTUD3 inhibitors. **A** Diagram of human OTUD3, showing ovarian tumor (OTU) and ubiquitin-associated (UBA) domains. **B** Crystal structure of the OTUD3 OTU domain. A cartoon representation is shown. The S1 Ub-binding site, N termini, and C termini are labeled. **C** Left: Crystal structure of the OTUD5 OTU domain (green) bound to Ub (orange) (PDB-ID:3TMP). Middle: Structure of the OTUD3 OTU domain (blue) superimposed to the OTUD5 OTU domain (green) bound to Ub (orange). Right: Approximate structure of the OTUD3 OTU domain (blue) bound to Ub (orange), Ub position was determined by sequence and structural alignment with OTUD5. **D** Identification of the docking GridBox that encloses the S1 Ub-binding site. **E** Schematic diagram of computational virtual screening based on molecular docking. **F** Cell proliferation assays showing that only compound 16 had a significant inhibitory effect on the proliferation of H1299 and H460 cell lines after 48 h of treatment. Data shown are mean ± SD. *n* = 5 independent experiments. Two-tailed unpaired Student’s *t*-test. ***p < 0.001, vs DMSO group. **G** Chemical structure of OTUDin3 (compound 16). **H** Surface representation of the structure of the OTUD3 OTU domain in complex with OTUDin3. The residues around OTUDin3 are highlighted in violet. **I** Overall structure of OTUD3 OTU domain in complex with OTUDin3, and Close-up view of the compound binding site highlighting key residues. OTUD3: skyblue cartoon; OTUDin3: yellow sticks; OTUD3 (Asn136, Gln154, Trp160, Ile162, Tyr177 and Tyr183) side chains are represented as sticks. Dashed lines showing the key interactions between OTUD3 and OTUDin3. Blue dashed lines represent hydrogen bonds. Green dashed lines indicate π-stacking interactions. Gray dashed lines indicate hydrophobic interactions. Binding site detail, showing the interactions between OTUDin3 and OTU domain residues. Hydrogen bonds are formed between Gln154, Trp160, Tyr177, Tyr183 and OTUDin3. π-stacking interactions are formed between Trp160, Tyr177 and OTUDin3. Hydrophobic interactions are formed between Asn136, Ile162 and OTUDin3. The PLIP package was used to analyze protein-ligand interactions. **J** Representative SPR sensorgram of OTUD3 OTU (aa 52-209) to measure affinity parameters of OTUDin3. The KD value was 0.32 μM. **K** OTUD3 OTU (aa 52-209) and K48-linked Di-Ubiquitin were incubated with indicated concentrations of OTUDin3 for 3 h at 37 °C, followed by western blotting with indicated antibodies. **L** Purified GST-OTUD3^C76A^ and Di-Ubiquitin was incubated with OTUDin3 or DMSO. Proteins retained on Sepharose were blotted with the indicated antibodies, or visualized by SDS-PAGE and Coomassie blue staining. **B**, **C**, **D**, **H**, and **I** The images were generated with The PyMOL Molecular Graphics System (version 2.6.0a0). **J**–**L** All panels are representative results of three or more independent experiments.

OTUD3 contains an ovarian tumor (OTU) domain and a ubiquitin-associated (UBA) domain (Fig. 1A). The OTU domain is the catalytic domain and is essential for OTUD3 to perform deubiquitinating activity and interact with substrates [23]. The OTUD3 (PDB: 4BOU) PDB file was downloaded from the Protein Data Bank (Fig. 1B). All of the heterogeneous atoms were removed, and the 4BOU chain A was selected for subsequent molecular docking. Previously developed inhibitors of DUBs have been found to bind the evolutionarily conserved catalytic centers of DUBs, resulting in poor selectivity [37]. However, a few inhibitors, such as the USP14 inhibitors IU1- series and USP7 inhibitor FT671, blocked access of the C-terminus of ubiquitin to the active site and showed surprisingly excellent selectivity [18, 21, 22]. Therefore, the S1 Ub-binding site was determined as the ligand binding site, which is key for the deubiquitinating activity of OTUD3 [38]. However, the structure of OTUD3 in complex with Ub was not yet resolved. Considering that the structure of OTUD3 is highly similar to OTUD5 with a low root mean square deviation (~0.8 Å), we performed superimposition of the OTUD3 OTU structure with the structure of OTUD5 OTU-Ub complex to identify the Ub-binding site of OTUD3 (Fig. 1C, Supplementary Fig. 1A). Then, the docking GridBox was set to enclose the entire S1 Ub-binding site and all the amino acid residues around it (Fig. 1D). AutoDock Vina 1.1.2 was used for the subsequent molecular docking [39]. A diverse set of 100 000 compounds available at ChemDiv was screened, and 18 candidate hits with the highest docking scores were identified for further biological experimental verification (Fig. 1E, Supplementary Table 1).

As OTUD3 plays a promoting role in lung tumorigenesis by stabilizing GRP78, inhibitors of OTUD3 should inhibit the proliferation of NSCLC cells [26]. Therefore, cell proliferation assays were chosen as a further screening strategy for the OTUD3 inhibitors. From this work, compound 16 was identified, which significantly inhibited the proliferation of H1299 cells after incubation with 10 μM of the candidate compound for 24 and 48 h (Fig. 1F, Supplementary Fig. 1B). The results were confirmed in H460 and A549 cell lines (Fig. 1F, Supplementary Fig. 1C).

We next performed molecular docking of compound 16, 4-[dioxo(1,2,3,4-tetrahydroisoquinolin-2-yl)-λ6-sulfanyl]-N-[3-(1H-benzo[d]imidazol-2-yl)phenyl]benzamide, referred to hereafter as OTUDin3 (Fig. 1G). Molecular docking analysis showed that the affinity energy of OTUDin3 to OTUD3 was −10.1 kcal/mol, and OTUDin3 was docked into the S1 Ub-binding site pocket (Fig. 1H). The predicted protein-ligand interactions mode showed that OTUDin3 formed multiple interactions with OTUD3, including hydrophobic interactions with Asn136 and Ile162, hydrogen bonds with Gln154, Trp160, Tyr177, and Tyr183, and π-stacking bonds with Trp160 and Tyr177 (Fig. 1I, Supplementary Fig. 1D).

To further confirm that OTUDin3 is an inhibitor of OTUD3, we first performed surface plasma resonance (SRP) assays. The results showed that OTUDin3 bound to OTUD3 with a dissociation equilibrium constant (KD) value of 0.32 μM (Fig. 1J). To explore the specificity of OTUDin3, OTUD5 was selected to verify whether OTUDin3 binds to other DUB. The results of SPR showed that OTUDin3 did not bind to OTUD5 (Supplementary Fig. 1E). This implied that OTUDin3 bound to OTUD3 with a certain specificity.

Next, we performed in vitro deubiquitination assays to verify whether OTUDin3 inhibits the deubiquitinating activity of OTUD3. The results indicated that OTUDin3 inhibited the deubiquitinating activity of OTUD3 to cleave K48- and K63- linked diUb (Fig. 1K, Supplementary Fig. 1F). Then, we performed in vitro OTUD3-Ub binding assays with OTUDin3 to verify whether OTUDin3 interfered with OTUD3 binding to Ub as designed. The results showed that OTUDin3 inhibited the binding of OTUD3 to Ub (Fig. 1L).

Collectively, we identified OTUDin3 as an inhibitor of OTUD3 using virtual screening and biological experiments, which represented a potentially tractable starting point suitable for further investigation.

### OTUDin3 inhibits NSCLC cell growth, migration and invasion

**Fig. 2:**
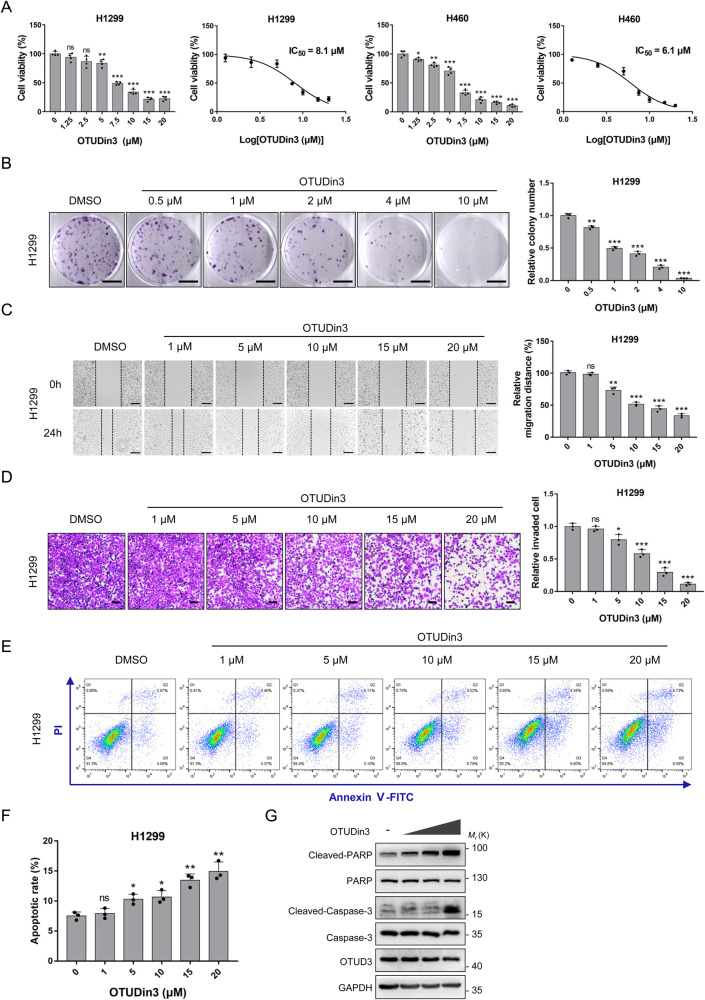
OTUDin3 inhibits lung cancer cell growth, migration and invasion, and induces apoptosis. **A** Cell proliferation assays in H1299 or H460 cells treated with increasing concentrations of OTUDin3 for 72 h. Viability of cells was determined by the CCK-8 kit. IC_50_ was analyzed by nonlinear regression (curve fit) using GraphPad Prism V7.0 software. **B** Representative images of colony formation assays in H1299 cells treated with increasing concentrations of OTUDin3 for 2 weeks after seeding of 1000 cells in six-well plate. Scale bar: 1 cm. **C** H1299 cells were wounded and then cultured in medium containing increasing concentrations of OTUDin3 for 24 h. Representative images of wound-healing assay were showed. Scale bar, 100 μm. **D** Representative images of invasion assays of H1299 cells treated with increasing concentrations of OTUDin3. Scale bar: 100 μm. **E**, **F** H1299 cells were treated with increasing concentrations of OTUDin3 for 24 h. The apoptotic cell population including early and late apoptosis cells was quantified using an apoptotic kit and analyzed by flow cytometry. The data were analyzed by FlowJo_V10.8.1 software (Treestar, Ashland, OR). The column chart (**F**) shows the quantification of apoptotic rate of H1299 cells after OTUDin3 treatment. **G** Western blotting analyses of cell lysates were performed with antibodies against Cleaved-PARP, Cleaved-Caspase-3, PARP, Caspase-3, OTUD3, and GAPDH, as indicated. A representative image of three independent experiments is shown. All the data shown are mean ± SD. *n* = 3 or 4 independent experiments. Two-tailed unpaired Student’s *t*-test. *p < 0.05, **p < 0.01, ***p < 0.001, ns = no significance, vs control group.

From preliminary screening, we found that OTUDin3 inhibited NSCLC cells at a concentration of 10 μM. Next, we used Cell Counting Kit 8 (CCK-8) assays to determine the 50% inhibitory concentration (IC_50_) of OTUDin3. The results showed that the IC_50_ value of OTUDin3 in H1299, A549, H460, and H1650 cells was 8.1, 14.8, 6.1, and 7.4 μM, respectively (Fig. 2A, Supplementary Fig. 2A). Microscopic images of the cell morphology showed that OTUDin3 induced cell death in H1299 cells (Supplementary Fig. 2B). Next, we detected the effects of OTUDin3 on normal cells, human pulmonary alveolar epithelial cells (HPAEpiC). The results showed that OTUDin3 had less inhibition on HPAEpiC cells, and the IC_50_ value was 102.3 μM which is an order of magnitude compared with lung cancer cells (Supplementary Fig. 2C). In addition, colony formation assays showed that OTUDin3 inhibited H1299 and A549 cell growth in a concentration-dependent manner (Fig. 2B, Supplementary Fig. 2D). To confirm whether OTUDin3 inhibited the migration of lung cancer cells, we performed wound-healing assays. The results showed that treatment with OTUDin3 significantly decreased the migration ability of H1299 cells in a concentration-dependent manner (Fig. 2C). Furthermore, OTUDin3 impaired the invasive ability of H1299 cells in a concentration-dependent manner, as assessed by Transwell assays (Fig. 2D). Collectively, these results demonstrated that OTUDin3 inhibited NSCLC cell growth, migration and invasion.

### OTUDin3 induces NSCLC cell apoptosis

We next evaluated the ability of OTUDin3 to induce apoptosis. H1299 cells were treated with increasing concentrations of OTUDin3. The apoptotic cell population, including early and late apoptotic cells, was quantified using an apoptosis kit and analyzed by flow cytometry. The results showed that OTUDin3 induced apoptosis in H1299 cells in a concentration-dependent manner (Fig. 2E, F). Cell lysates were assessed by western blotting. Consistently, OTUDin3 increased the levels of apoptosis-associated proteins, Cleaved poly (ADP-ribose) polymerase (PARP) and Cleaved Caspase-3, in a concentration-dependent manner (Fig. 2G). Together, the results indicated that OTUDin3 induced NSCLC cell apoptosis.

### OTUDin3 inhibits NSCLC cell growth, migration and invasion, and induces apoptosis by targeting OTUD3

**Fig. 3:**
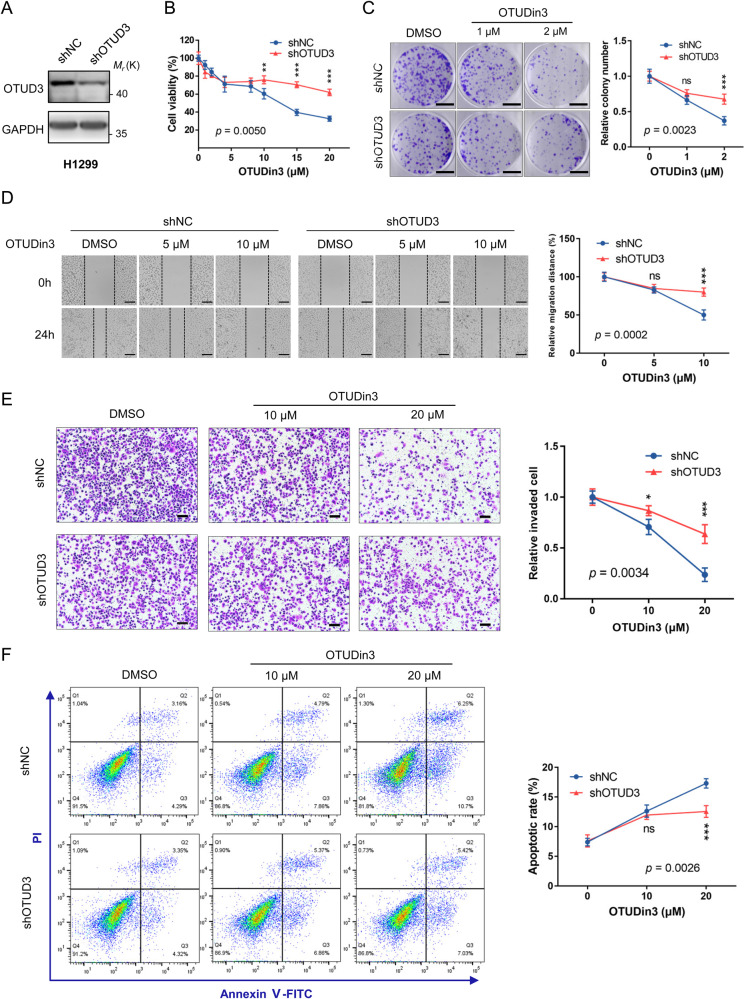
OTUDin3 inhibits NSCLC cell growth, migration and invasion, and induces apoptosis by targeting OTUD3. **A** *OTUD3* was knocked down by shRNA in H1299 cells. The protein levels of OTUD3 were analyzed by western blotting. **B** Cell proliferation assays in H1299 shNC and H1299 shOTUD3 cells treated with increasing concentrations of OTUDin3 for 72 h. Viability of cells was determined by the CCK-8 kit. **C** Representative images of colony formation assays in H1299 shNC and H1299 shOTUD3 cells treated with indicated concentrations of OTUDin3. Scale bar: 1 cm. **D** Representative images of wound-healing assay in H1299 shNC and H1299 shOTUD3 cells. Cells were wounded and then incubated with indicated concentrations of OTUDin3 for 24 h. Scale bar, 100 μm. **E** Representative images of invasion assays in H1299 shNC and H1299 shOTUD3 cells treated with indicated concentrations of OTUDin3. Scale bar: 100 μm. **F** H1299 shNC and H1299 shOTUD3 cells were treated with indicated concentrations of OTUDin3 for 24 h. The apoptotic cell population including early and late apoptotic cells was quantified using an apoptotic kit and analyzed by flow cytometry. The data were analyzed by FlowJo_V10.8.1 software (Treestar, Ashland, OR). All the data shown are mean ± SD. *n* = 3 or 5 independent experiments. Two-sided *P*-values were calculated using two-way ANOVA followed by Fisher’s least significant difference test. *p < 0.05, **p < 0.01, ***p < 0.001, ns = no significance, H1299 shNC vs H1299 shOTUD3.

To investigate whether the effects on NSCLC cells were mainly derived from targeting OTUD3, we knocked down *OTUD3* in H1299 cells (Fig. 3A). Then, using the *OTUD3* knockdown cells, we performed the same series of experiments previously performed. The CCK-8 assay results showed that compared with the control, the inhibitory effect on cell proliferation was significantly reduced in *OTUD3* knockdown cells (Fig. 3B). Similarly, OTUDin3 had a weaker impact on colony formation in *OTUD3* knockdown cells (Fig. 3C). Wound healing and Transwell assays showed that *OTUD3* knockdown cells were less sensitive to OTUDin3 compared with the control (Fig. 3D, E). The results of apoptosis assay showed that *OTUD3* knockdown cells were less responsive to OTUDin3, which was consistent with the above results (Fig. 3F, Supplementary Fig. 3A). However, we observed that OTUDin3 still inhibited cell growth after *OTUD3* knockdown. We speculated that it may be due to low knockdown efficiency. Therefore, we constructed *OTUD3* knockout (KO) cell line using the CRISPR/Cas9 technology (Supplementary Fig. 3B), and then performed CCK-8 assays. The results showed that OTUDin3 had less inhibitory effect on the *OTUD3* KO cells than on the *OTUD3* knockdown cells (Supplementary Fig. 3C). While OTUDin3 inhibited about 50% of *OTUD3* wild-type (WT) cells at a concentration of 10 μM, it had little inhibitory effect on *OTUD3* KO cells (Supplementary Fig. 3C). Taken together, these results demonstrated that OTUDin3 inhibited lung cancer cell growth, migration and invasion, and induced apoptosis by targeting OTUD3.

### OTUDin3 downregulates GRP78 by inhibiting the deubiquitinating activity of OTUD3

**Fig. 4:**
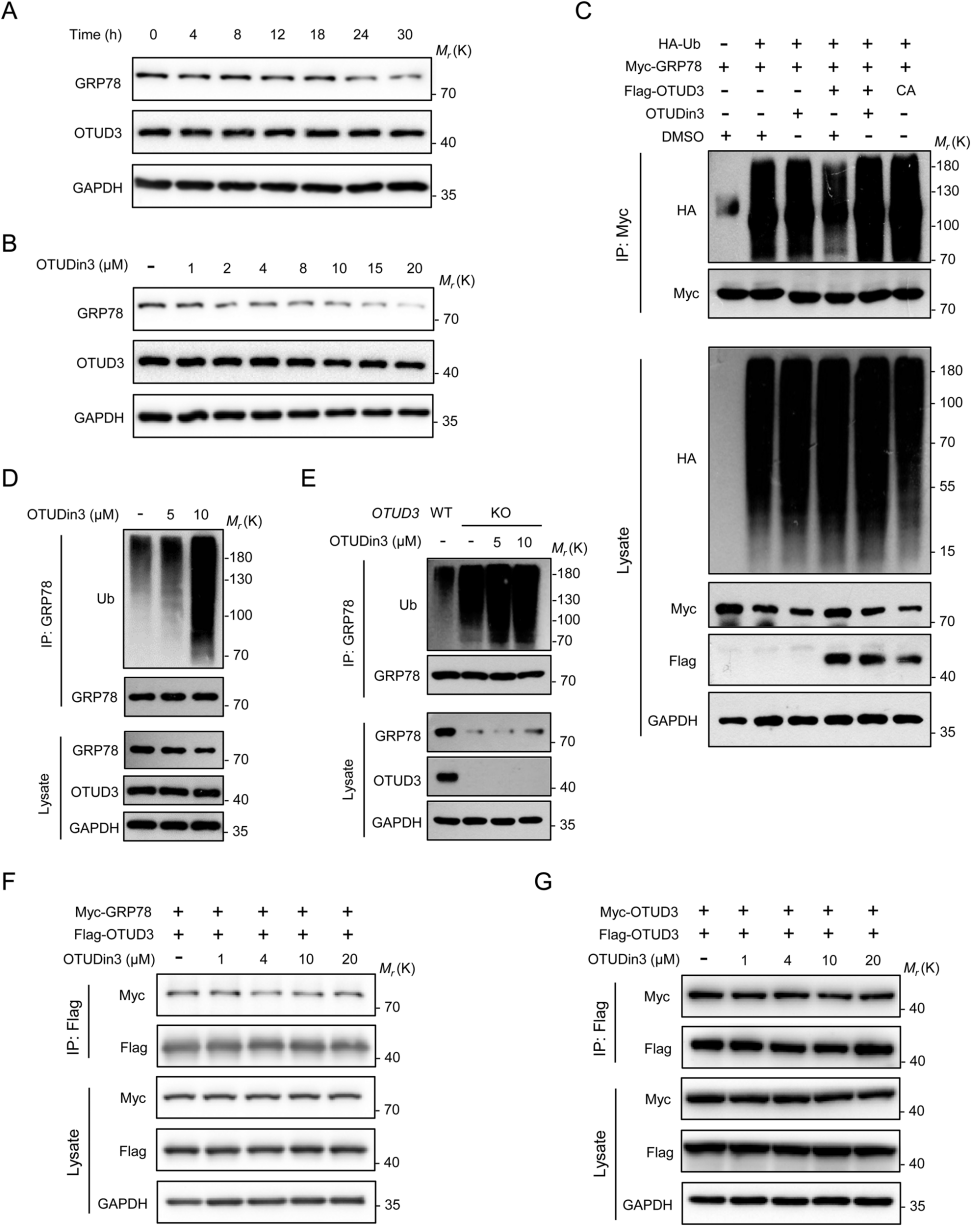
OTUDin3 downregulates GRP78 by inhibiting the deubiquitinating activity of OTUD3. **A** H1299 cells treated with 5 μM OTUDin3 for the indicated time were lysed for western blotting analysis probing for GRP78, OTUD3, and GAPDH as indicated. **B** H1299 cells were treated with increasing concentrations of OTUDin3 for 24 h, and cell lysates were assessed by western blotting. **C** HEK293T cells were transfected with HA-Ub, Myc-GRP78, Flag-OTUD3^WT^, Flag-OTUD3^C76A^ alone or in combination and incubated with or without 5 μM OTUDin3 for 46 h. Proteasome inhibitor (20 μM MG132) were added 6 h before lysis. Cell lysates were immunoprecipitated with anti-Myc antibody, followed by western blotting with indicated antibodies. **D** H1299 cells were treated with indicated concentrations of OTUDin3 for 40 h. Proteasome inhibitor (20 μM MG132) were added 8 h before lysis. Cell lysates were subjected to immunoprecipitation with anti-GRP78 antibody, followed by western blotting with indicated antibodies. **E** *OTUD3* KO H1299 cells were treated with indicated concentrations of OTUDin3 for 40 h. Proteasome inhibitor (20 μM MG132) were added 8 h before lysis. Cell lysates were subjected to immunoprecipitation with anti-GRP78 antibody, followed by western blotting with indicated antibodies. **F** Flag-OTUD3 and Myc-GRP78 were co-transfected into HEK293T cells with indicated concentrations of OTUDin3 for 24 h. Cell lysates were subjected to immunoprecipitation with anti-Flag antibody, followed by western blotting with indicated antibodies. **G** Flag-OTUD3 and Myc-OTUD3 were co-transfected into HEK293T cells with indicated concentrations of OTUDin3 for 24 h. Cell lysates were subjected to immunoprecipitation with anti-Flag antibody, followed by western blotting with indicated antibodies. All panels are representative results of three or more independent experiments.

We then investigated the underlying mechanisms by which OTUDin3 inhibited NSCLC cells. A previous study demonstrated that OTUD3 promotes lung tumorigenesis by stabilizing GRP78 [26]. Therefore, we performed a time-course analysis using western blotting to check the protein level of GRP78. H1299 cells were treated with 5 μM of OTUDin3 for the indicated time and then lysed. Western blotting analysis showed that OTUDin3 downregulated GRP78 in a time-dependent manner, and the downregulation effect was significant after 24 h (Fig. 4A). Then, H1299 cells were treated with increasing concentrations of OTUDin3 for 24 h. Western blotting analysis showed that OTUDin3 downregulated GRP78 in a concentration-dependent manner without affecting the stability of OTUD3 (Fig. 4B). The result was consistent with the downregulation of GRP78 by *OTUD3* knockdown in the H1299 cell line [26].

OTUD3 upregulates GRP78 in a manner dependent on its deubiquitinating activity, as the catalytically inactive mutant C76A lost its ability to upregulate GRP78 [26]. To verify the downregulation effect of OTUDin3 on GRP78 resulting from inhibition of the deubiquitinating activity of OTUD3, GRP78 ubiquitylation assays were performed. We first performed the ubiquitination assay of exogenous GRP78 in HEK293T cells. Flag-OTUD3^WT^ or Flag-OTUD3^C76A^, Myc-GRP78, and HA-Ub were transfected into HEK293T cells as indicated. The different groups were treated with 5 μM of OTUDin3 or an equal volume of dimethyl sulfoxide (DMSO) for 46 h. The results showed that overexpression of OTUD3^WT^ significantly reduced the level of GRP78 ubiquitination, and the catalytically inactive mutant OTUD3^C76A^ did not reduce the level of GRP78 ubiquitination. However, when OTUD3^WT^ overexpressing cells were treated with OTUDin3, GRP78 ubiquitination was increased, reversing the deubiquitination of OTUD3 (Fig. 4C). The results indicated that OTUDin3 increased GRP78 ubiquitylation by inhibiting the deubiquitinating activity of OTUD3. Then, endogenous GRP78 ubiquitination assays were performed in H1299 cells to further confirm the above mechanism. The results showed that OTUDin3 increased GRP78 ubiquitylation in a concentration-dependent manner (Fig. 4D). To verify that the increased GRP78 ubiquitination by OTUDin3 was due to targeting OTUD3, we performed endogenous GRP78 ubiquitination assays in *OTUD3* KO cells. The results showed that OTUDin3 had little effect on GRP78 ubiquitination in *OTUD3* KO cells (Fig. 4E). Collectively, OTUDin3 increased GRP78 ubiquitylation by inhibiting the deubiquitinating activity of OTUD3.

A previous study showed that the N-terminal OTU domain of OTUD3 mediated the physical interaction with GRP78 [26]. Given that OTUDin3 binds to the OTU domain, we further investigated whether OTUDin3 disrupted the interaction between OTUD3 and GRP78 by performing co-IP and GST-pulldown assays. Co-IP and GST-pulldown assays confirmed that OTUDin3 did not affect the interaction between OTUD3 and GRP78 (Fig. 4F, Supplementary Fig. 4A). In addition, OTUD3 formed homodimers to perform deubiquitinating activity [23]. Co-IP and GST pull-down assays showed that OTUDin3 also did not affect the dimerization of OTUD3 (Fig. 4G, Supplementary Fig. 4B).

Taken together, OTUDin3 enhanced degradation of GRP78 by inhibiting the deubiquitinating activity of OTUD3 and increasing GRP78 ubiquitylation. These results are consistent with the expected mechanism of an OTUD3 inhibitor.

### OTUDin3 inhibits NSCLC growth in vivo

**Fig. 5:**
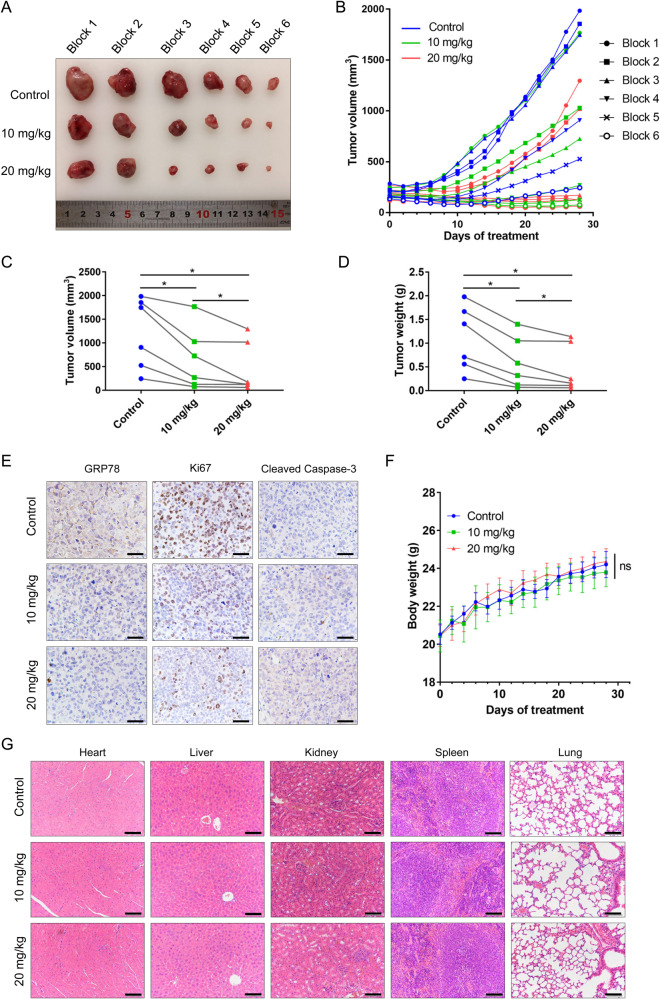
OTUDin3 inhibits the NSCLC growth in vivo. **A** Photographic images of xenograft tumors from control and OTUDin3 treatment groups. Tumor-bearing mice were divided into 6 blocks according to tumor size, and then mice in each block were randomly assigned to different groups (Randomized block design). **B** The growth curves of xenograft tumors in each mouse are shown, the length and width of tumors were measured every other day. Randomized block design. Tumor volume was calculated by the formula: *V* = 0.5 × length × width^2^. **C** Tumor growth was significantly reduced in a dose-dependent manner by treatment with OTUDin3. The volumes of harvested xenograft tumors were measured. Randomized block design. Wilcoxon test. *p < 0.05. **D** The weights of harvested xenograft tumors were measured. Randomized block design. Wilcoxon test. *p < 0.05. **E** Representative immunohistochemical staining images of GRP78, Ki67 and Cleaved Caspase-3 in tumors from nude mice with or without OTUDin3 treatment. Scale bar, 50 µm. **F** Analysis of body weight during treatment of H1299 xenograft-bearing mice with OTUDin3. The data shown are mean ± SD, *n* = 6, Two-way ANOVA, ns = no significance. **G** Representative H&E staining images of hearts, livers, kidneys, lungs and spleens from nude mice with or without OTUDin3 treatment. Scale bar, 100 μm.

To further investigate the effects of OTUDin3 on NSCLC in vivo, we next performed an H1299 xenograft mouse model experiment in which nude mice were implanted with H1299 cells. When tumors reached ~150–250 mm^3^, 18 tumor-bearing mice were divided into 6 blocks according to tumor size, and then the mice in each block were randomly assigned to different groups. The treatment groups were administered 10 mg/kg and 20 mg/kg of OTUDin3 by intraperitoneal injection every other day, respectively. Under identical experimental conditions, the control group was administered corresponding volume of solvent. The results showed that treatment with OTUDin3 led to a significant inhibition of tumor growth (Fig. 5A). Compared with the control group, tumor volume and weight were significantly reduced in the treatment groups (Fig. 5B-D). Immunohistochemical results showed that compared with the control group, the levels of GRP78 and Ki67 in the treatment groups decreased, and the level of Cleaved Caspase-3 increased (Fig. 5E). Meanwhile, there was no significant difference in average body weight between the control and treatment groups (Fig. 5F). Concomitantly, H&E staining showed that OTUDin3 had no significant effect on the major organs including heart, liver, spleen, lung and kidney (Fig. 5G). Summarizing, OTUDin3 inhibited the NSCLC growth in vivo with no obvious health problems or weight loss of mice.

## Discussion

As critical components in the regulation of protein homeostasis, DUBs are extensively involved in cell cycle regulation, DNA damage repair and cell growth control, which are all hallmarks of tumorigenesis [9]. Mechanistically, most cancers are characterized by overexpression of oncoproteins, which are normally degraded by the ubiquitin-proteasome pathway. However, when DUBs are aberrantly activated genetically or functionally, degradation of their corresponding oncoprotein substrates may be reduced, leading to oncoprotein accumulation [40]. Therefore, inhibition of the aberrantly activated DUBs could be a novel and promising anti-cancer strategy. Currently, an increasing number of potent inhibitors targeting oncogenic DUBs have been developed [37]. A variety of DUB inhibitors have been verified to be effective in malignant tumors, especially those targeting USP1, USP7, and UPS14, displaying great potential for clinical application, such as ML323, SJB3-019A, KSQ-4279, Compound 4, FT671 and VLX1570 [14, 16–18, 41, 42]. Most encouragingly, KSQ-4279, a first-in-class USP1 inhibitor, has entered a Phase I clinical study for patients with advanced solid tumors (NCT05240898). In the meanwhile, the number of inhibitors targeting non-USP DUBs is limited. Although the research and development of DUB inhibitors has made great progress in recent years, it is still in its infancy, and the DUB inhibitor field still has great potential for drug development.

The deubiquitylase OTUD3 was initially demonstrated to stabilize the tumor suppressor PTEN and play a suppressive role in breast tumorigenesis [23]. However, further study showed that *Otud3* transgenic mice were more susceptible to Kras^G12D^-driven lung cancer while *Otud3* KO mice were less susceptible, implicating an oncogenic role of OTUD3 in lung cancer [26]. Meanwhile, OTUD3 was found to be highly expressed in human NSCLC tissues, and the high expression of OTUD3 correlated with poor survival in NSCLC patients [26]. Mechanistic studies indicated that OTUD3 promoted lung tumorigenesis by deubiquitylating and stabilizing GRP78 [26]. GRP78 is a potent anti-apoptotic protein and plays a vital role in tumor cell survival, tumor progression and angiogenesis, metastasis, and resistance to therapy [43]. In addition, a study on CHIP, an E3 ubiquitin ligase for OTUD3, further confirmed that OTUD3 promoted NSCLC through an OTUD3-GRP78 signaling axis [44]. Thus, OTUD3 is a potential drug target for anti-cancer therapy in NSCLC. In addition, *Otud3* KO in mice did not cause strong adverse effects, such as lethality or developmental defects [23]. Taken together, inhibition targeting OTUD3 is feasible and safe.

In this study, we identified lead compounds using virtual screening against a diverse set of 100 000 compounds. Virtual screening technology is the core of CADD. With the rapid increase in the availability of protein and small-molecule databases, the applicability of CADD has been extended to nearly every stage of drug discovery, including target identification and validation, lead discovery and optimization and preclinical tests [45]. Compared with traditional methods, CADD increases the hit rate of drug screening by >100 times, from ~0.01% to 1~2% [46]. Due to the high hit rates, CADD is becoming the fundamental basis of industrial drug discovery as well as academic research [47]. Currently, DUB inhibitors were identified mainly based on HTS, and the most frequently used substrates for DUB activity profiling are ubiquitin-7-amino-4-methylcoumarin (Ub-AMC) and more recently Ub-rhodamine (Ub-Rho) [12]. Compared with HTS, virtual screening is more efficient and economical, which is an alternative strategy for inhibitors discovery of DUBs with known crystal structures.

**Fig. 6:**
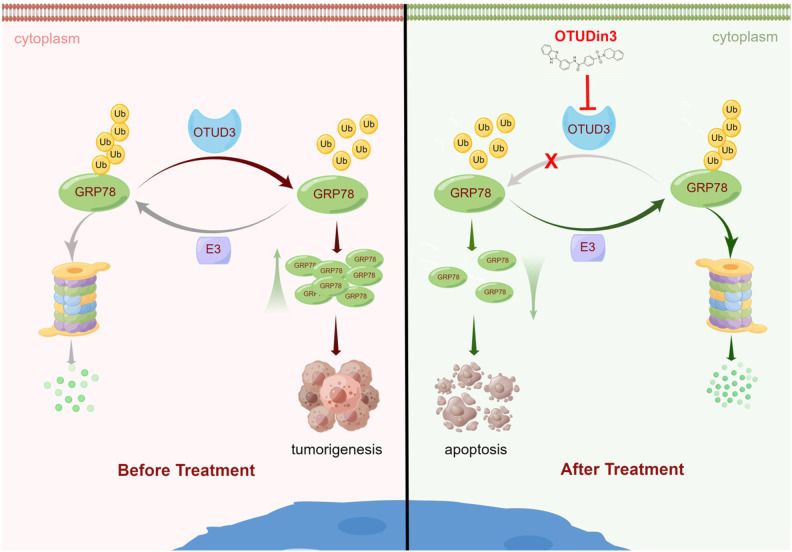
Model of OTUDin3 inhibits NSCLC through targeting deubiquitylase OTUD3. OTUDin3 inhibits the deubiquitinating activity of OTUD3, resulting in enhanced ubiquitination of GRP78 and a consequent decrease in GRP78 protein levels, thereby inducing apoptosis of NSCLC cells. By Figdraw.

After virtual screening and biological experiments, we identified OTUDin3 as an OTUD3 inhibitor. Then, we demonstrated that OTUDin3 inhibited NSCLC cell growth, migration and invasion, and induced apoptosis by inhibiting the deubiquitinating activity of OTUD3 and enhancing the ubiquitination and degradation of GRP78. Finally, an H1299 xenograft mouse model experiment also demonstrated that OTUDin3 inhibited lung tumor growth in vivo by downregulating the level of GRP78. Based on these findings, we concluded that OTUDin3 inhibits NSCLC by targeting the OTUD3-GRP78 signaling axis (Fig. 6). Identification of OTUDin3 confirmed that targeting DUBs is a viable strategy for anti-cancer therapy. Our findings are encouraging, and further studies aimed at resolving the co-crystal structure of OTUD3 complexed with OTUDin3 and optimization of OTUDin3 for further investigation are warranted and will be reported at a later stage.

OTUD3 plays context-dependent roles in tumorigenesis of different types of cancers [48]. In addition to regulating GRP78 to promote tumorigenesis in NSCLC, OTUD3 suppressed tumorigenesis in breast, colon, liver, brain, esophageal, and cervical cancer by stabilizing PTEN, ZFP36 or p53 [23–25]. Recently, a study showed that OTUD3 is an important regulator of glucose and lipid metabolism, and OTUD3 insufficiency is associated with obesity and a higher risk of diabetes [27]. In addition, OTUD3 prevented Parkinson’s disease (PD) by stabilizing iron regulatory protein 2 [28]. Depletion of OTUD3 resulted in a disorder of iron metabolism, motor deficits, and nigrostriatal dopaminergic neurodegeneration, which resembled the pathology of PD [28]. Collectively, OTUD3 plays a positive role in these physiological processes, which means that an OTUD3 inhibitor may cause corresponding side effects. Therefore, it is necessary to consider drug delivery technology in the application of OTUD3 inhibitors in NSCLC treatment. At present, the research on targeted drug delivery for lung cancer has made progress [49]. The application of a targeted drug delivery system can be considered to avoid possible side effects of OTUDin3.

The good news is that OTUD3 plays a negative role in innate antiviral immunity via restricting innate antiviral immune signaling [50]. OTUD3 deficiency in mice results in enhanced innate immunity, a diminished viral load, and morbidity [50]. This implies that OTUD3 may have potential application in antiviral therapy.

In summary, we identified the first inhibitor of OTUD3 by virtual screening and biological experiments, which exhibited potent NSCLC inhibition both in vitro and in vivo. OTUDin3 enhanced degradation of the substrate GRP78 by inhibiting the deubiquitinating activity of OTUD3. Our findings suggest that inhibition targeting OTUD3 may be a promising therapeutic strategy for NSCLC.

## Materials and methods

### Computational virtual screening

The three-dimensional structures of OTUD3 (PDB ID: 4BOU) and OTUD5 (PDB ID: 3TMP) were obtained from the Protein Data Bank (http://www.rcsb.org/). The structure of OTUD3 was prepared before molecular docking using AutoDockTools-1.5.7. The GridBox was defined to include S1 Ub binding site. A diversity set of 100 000 Screening Compounds in SDF format were obtained from the ChemDiv (http://www.chemdiv.org/). The SDF format files were converted to PDB format using Open Babel 3.1.1. The converted PDB files were then converted to PDBQT format using a ligand preparation script from AutoDockTools-1.5.7. Then, AutoDock Vina 1.1.2 was used for the subsequent molecular docking. Detailed procedures are described in the protocol [51]. The small molecules were ranked based on the affinity energy.

All of the visualization of the structure files was performed using PyMOL Molecular Graphics System, Version 2.6.0a Schrödinger, LLC. Protein–ligand interactions were analyzed using the Protein-Ligand Interaction Profiler web tool.

### Cell lines and culture conditions

The cell lines H1299, H1650, H460, A549, HPAEpiC and HEK293T were obtained from the American Type Culture Collection (ATCC), authenticated by STR profiling (Promega) and shown to be mycoplasma-free using a mycoplasma test. H1299, H1650, H460 and HPAEpiC cells were cultured in Roswell Park Memorial Institute (RPMI) 1640 medium supplemented with 10% fetal bovine serum (FBS) and 1% penicillin-streptomycin. A549 and HEK293T cells were cultured in DMEM supplemented with 10% FBS and 1% penicillin-streptomycin. All of the cells were grown in a humidified environment with 5% CO_2_ at 37 °C. Medium and supplements were purchased from Gibco except where indicated.

### Reagents, recombinant proteins and antibodies

OTUDin3 was purchased from ChemDiv (G856-0029). OTUD3 OTU (aa 52-209) and OTUD5 OTU (aa 168-351) recombinant proteins were purchased from SinoBiological. K48-linked Di-Ubiquitin (P20022) and K63-linked Di-Ubiquitin (P20023) were purchased from Solarbio. Antibodies used in the study were anti-GAPDH (1:1000, AC033, ABclonal), anti-OTUD3 (1:500, HPA028543, Sigma), anti-Cleaved-PARP (1:1000, #9544, Cells Signaling Technology), anti-Cleaved-Caspase-3 (1:500, #9661, Cells Signaling Technology), anti-PARP (1:1000, #9532, Cells Signaling Technology), anti-Caspase-3 (1:1000, #9662, Cells Signaling Technology), anti-Ubiquitin (1:1000, #58395, Cells Signaling Technology), anti-GRP78 (1:1000, 11587-1-AP, Proteintech), anti-HA (1:1000, M180-3, MBL), anti-Myc (1:1000, M192-3, MBL), and anti-Flag (1:1000, F7425, Sigma).

### Surface plasmon resonance (SPR) assays

SPR data were acquired on Biacore T200 instruments (Cytiva) at room temperature. CM5 Sensor Chip (Cytiva) was activated by using sulpho-NHS/EDC chemistry in a buffer consisting of 2.7 mM KCl, 137 mM NaCl, 0.05% (v/v) surfactant P20, pH 7.4. The chip was subsequently immobilized with the target proteins at a concentration of 50 ug/mL in sodium acetate, pH 4.0 for OTUD3 OTU (aa 52-209) and blocked with 1 M ethanolamine, pH 8.0. The proteins immoblized on CM5 chip reached target densities of 6000 resonance units (RU). Ligands were dissolved to 100 μM in 100% DMSO and then 20-fold into running buffer without DMSO then diluted 2-fold by 5% DMSO into 5 μM, 1.25 μM, 0.625 μM, 0.3125 μM, 0.156 μM, 0.078 μM and 0 μM before injection. The running buffer contained previous running buffer and 5% DMSO. Data were analyzed with Biacore T200 Evaluation Software and GraphPad Prism.

### In vitro deubiquitination assays

Use the purified OTUD3 OTU (aa 52-209) and Di-Ubiquitin for the in vitro deubiquitination assay. Add 2 μg of OTUD3 OTU (aa 52-209) and 2 μg of Di-Ubiquitin to 40 μL buffer (20 mM HEPES, pH 7.2, 150 mM NaCl, and 10 mM DTT). Then, OTUDin3 or DMSO were added to the different groups. Carry out the deubiquitination reaction for 3 h at 37 °C with occasional shaking. Stop the in vitro reaction by adding 40 μL of 2 × loading buffer and analyze by western blotting.

### In vitro ubiquitin and DUB binding assays

Bacterially expressed GST-OTUD3^C76A^ bound to glutathione-Sepharose 4B beads (from GE) were incubated with Di-Ubiquitin for 8 h at 4 °C in the presence of DMSO or OTUDin3. Then the beads were washed with HEPES buffer four times. Add 40 μL HEPES and equal volume 2× loading buffer, and analyze by western blotting or Coomassie blue staining.

### Plasmids

Full-length OTUD3 WT and full-length OTUD3 C76A mutant were cloned into the pFlag-CMV-2 vector. Full-length OTUD3 WT and full-length GRP78 WT were cloned into the pCMV-Myc vector. GST-OTUD3 and GST-OTUD3^C76A^ were cloned into the pGEX-4T-2 vector. OTUD3 CRISPR/Cas9 KO (sc-407066) and OTUD3 HDR (sc-407066-HDR) plasmids were purchased from Santa Cruz Biotechnology.

### Lentivirus infection

To generate the lentiviral shRNA constructs against human *OTUD3*, the *OTUD3* shRNA sequences were cloned into the pCDH-puro vector. The shRNA sequences were reported in previous research [26]. The viruses were used to infect cells in the presence of polybrene. Forty-eight hours later, H1299 cells were cultured in medium containing puromycin for the selection of stable clones. The clones that stably knocked down *OTUD3* were identified and verified by western blotting. The *OTUD3* shRNA sequence was: 5′-TGGAAATCAGGGCTTAAAT-3′.

### Cell viability assays

Cells were seeded in a 96-well plate at a density of 5000 cells per well. Twenty-four hours later, cells were treated with DMSO or the indicated compounds at varying concentrations for 72 h, and then assessed for viability using a CCK-8 Kit (CK001-500T, LABLEAD). For the CCK-8 assay, 10 μL of CCK-8 which was dissolved in 90 μL of medium was added to each well. The absorbance at 450 nm was measured by a microplate reader (PerkinElmer) after being incubated at 37 °C for 1 h. IC_50_ values were obtained using GraphPad Prism with nonlinear regression.

### Colony formation assays

The cells were diluted to a single-cell suspension and 1000 cells were cultured in each well of a six-well plate at 37 °C with 5% CO_2_ incubator for 24 h. Then cells were treated with different concentrations of OTUDin3 for 10–14 days until clones were clearly visible. Cell culture plates were gently washed by PBS twice, fixed with a 4% paraformaldehyde phosphate buffer for 15 min and then stained with crystal violet for 15 min. Washing the excess stain with ddH_2_O and drying them at room temperature for several hours. Photographs of the stained colonies were taken.

### Wound-healing assays

Wound-healing assays were performed to evaluate the mobility of the cells. Cells were seeded in 6-well plates. After 24 h of culture, each well was manually scratched with a 200 μL pipette tip, and washed with PBS three times. Then, the cells were cultured in basic RPMI-1640 medium without FBS, and treated with varying concentrations of OTUDin3. The scratch area was photographed 24 h later. The distance between two cell edges was analyzed using ImageJ software (NIH).

### Invasion assays

A cell invasion assays were performed in a 24-well Transwell plate with 8.0 µm polycarbonate membrane inserts (Corning) coated by 60 µL Matrigel matrix (Corning). In brief, 2 × 10^5^ H1299 cells resuspended in serum-free medium with varying concentration of OTUDin3 were plated in each insert. The inserts were placed in the well containing RPMI 1640 medium with 10% FBS. After 24 h, non-invaded cells were removed and the inserts were washed in PBS, fixed in 4% formaldehyde for 20 min and stained with 0.1% crystal violet for 20 min. The wells were photographed and the stained cells were counted.

### Flow cytometry-based apoptosis detection

Flow cytometry-based apoptosis assays were performed using an Annexin V-FITC/PI Apoptosis Detection kit (P04D02, Gene-Protein Link). H1299 cells in 6-well plates were treated with different concentrations of OTUDin3 for 24 h. The cells were collected by centrifugation and resuspended in 100 µL of binding buffer. Then, 5 µL of Annexin V-FITC and 5 µL of propidium iodide (PI) were added and incubated at room temperature away from light for 10 min. Then, 400 μL of binding buffer was added, and samples were detected by flow cytometry within 1 h. The data were processed by FlowJo_V3.8.1 software.

### *OTUD3* KO cell line construction

In a 6-well tissue culture plate seed 1.5 × 10^5^—2.5 × 10^5^ cells in 3 ml of standard growth medium per well, 24 h prior to transfection. Grow cells to a 40–60% confluency. Initial cell seeding and cell confluency after 24 h are determined based on the rate of cell growth of the cells used for transfection. Healthy and subconfluent cells are required for successful KO and HDR plasmid transfection. Co-transfect 2 μg of OTUD3 CRISPR/Cas9 KO Plasmid and 2 μg of HDR Plasmid into cells. Incubate the cells for 48 h under conditions normally used to culture the cells. No media replacement is necessary during the first 24 h post-transfection. Add or replace media as needed 24–48 h post-transfection. Forty-eight hours post-transfection, replace with fresh medium containing 2 µg/ml of puromycin. Select cells for a minimum of 3–5 days. Then, cells were lysed for western blotting to confirm *OTUD3* knockout.

### Western blotting

Western blotting analysis was performed using standard techniques. The cells were collected with RIPA lysis buffer (50 mM Tris-Cl pH 8.0, 150 mM NaCl, 1% NP-40, 0.5% sodium deoxycholate, and 0.1% SDS) added with phosphatase and protease inhibitors. Proteins were separated by SDS-PAGE and transferred to nitrocellulose filter (NC) membranes (Merck Millipore, Germany). The membranes were blocked with 5% skim milk for 1 h at room temperature and then incubated with the indicated antibody overnight. The membranes were washed three times with Tris-buffered saline with Tween (TBST) for 10 min each. The NC membranes were incubated with secondary antibodies for 1 h at room temperature and then washed with TBST four times for 10 min each. Finally, Using the FluorChem R Imaging System (ProteinSimple, USA) to detect the expression of target proteins.

### In vivo exogenous GRP78 ubiquitylation assays

For in vivo GRP78 ubiquitylation assays, Flag-OTUD3 or Flag-OTUD3^C76A^, Myc-GRP78 and HA-Ub were transfected into HEK293T cells. The different groups were treated with 5 μM of OTUDin3 or an equal volume of DMSO for 40 h. Cells were lysed with RIPA lysis buffer after treated with 20 µM of the proteasome inhibitor MG132 (Calbiochem) for 6 h and incubated with anti-Myc antibody for 3 h and protein A/G agarose beads (Santa Cruz) for a further 6 h at 4 °C. Then the beads were washed three times with RIPA buffer. The proteins were released from the beads by boiling in SDS–PAGE sample buffer and analyzed by immunoblotting with anti-HA monoclonal antibody (MBL).

### In vivo endogenous GRP78 ubiquitylation assays

The cells were treated with different concentrations of OTUDin3 or an equal volume of DMSO for 40 h. Cells were lysed with RIPA lysis buffer after treated with 20 µM of the proteasome inhibitor MG132 (Calbiochem) for 8 h and incubated with anti-GRP78 antibody for 6 h and protein A/G agarose beads (Santa Cruz) for a further 8 h at 4 °C. Then the beads were washed three times with RIPA buffer. The proteins were released from the beads by boiling in SDS-PAGE sample buffer and analyzed by western blotting.

### Co-immunoprecipitation assays

HEK293T cells were transfected with indicated plasmids using Polyethylenimine (PEI, Invitrogen) reagent according to the manufacturer’s protocol, and treated with DMSO or different concentrations of OTUDin3. For immunoprecipitation assays, cells were lysed with HEPES lysis buffer (20 mM HEPES, pH 7.2, 50 mM NaCl, 0.5% Triton X-100, 1 mM NaF and 1 mM dithiothreitol) supplemented with a protease-inhibitor cocktail (Roche). Immunoprecipitations were performed using the indicated primary antibody and protein A/G agarose beads (Santa Cruz) at 4 °C. The immunocomplexes were then washed with HEPES lysis buffer four times. Both lysates and immunoprecipitates were examined using the indicated primary antibodies followed by detection with the related secondary antibody and the SuperSignal West Pico chemiluminescence substrate (Thermo).

### GST pulldown assays

Bacterially expressed GST and GST-OTUD3 bound to glutathione-Sepharose 4B beads (from GE) were incubated with Myc-GRP78 or Flag-OTUD3 expressed in HEK293T cells for 4 h at 4 °C in the presence of DMSO or different concentrations of OTUDin3. Then the beads were washed with GST binding buffer (100 mM NaCl, 10 mM Tris, 50 mM NaF, 2 mM EDTA, 0.5 mM Na3VO4 and 1% NP40) four times and proteins were eluted, followed by western blotting or Coomassie blue staining.

### NSCLC xenograft study

The animal experimental protocols were approved by the Animal Care and Use Committee of Beijing Institute of Lifeomics, China. Male BALB/c nude mice (6–8 weeks old) purchased from Vital River Laboratory Animal Technology Co, were subcutaneously inoculated with 6 × 10^6^ H1299 tumor cells in 1:1 mixture of RPMI-1640 and Matrigel (Corning). The efficacy study was initiated when tumors reached ~150–250 mm^3^. Using randomized block design, 18 tumor-bearing mice were divided into 6 blocks according to tumor size, and then mice in each block were randomly assigned to different groups. Mice received a dosing regimen of 10 or 20 mg/kg OTUDin3 every other day (intraperitoneal injection), and 10% DMSO, 10% polyoxyethylene castor oil and 80% saline served as a vehicle control. Body weight and tumor volume of the mice were monitored every other day. The length and width of tumors were measured with a vernier caliper. Tumor volume was calculated by the formula: *V* = 0.5 × length × width^2^. When the maximum tumor volume of mice reached 2000 mm^3^, tumor samples were collected. H&E staining and immunohistochemistry were performed by Wuhan Servicebio Technology Co., Ltd. All animals were handled in strict accordance with the “Guide for the Care and Use of Laboratory Animals” and the “Principles for the Utilization and Care of Vertebrate Animals”, and all of the animal work was approved by the Institutional Animal Care and Use Committee at the Beijing Institute of Lifeomics. Studies were tailored to minimize the number of animals used, yet allow sufficient numbers to address any variability in drug exposure. For efficacy studies where growth rate differences introduce some variability, *n* = 6 engrafted animals per treatment cohort provided sufficient statistical power for a well-behaved xenograft model. Owing to the need to monitor potential adverse effects with first in-life assessment of novel compounds, no study was conducted under blinded conditions.

### Statistical analysis

Statistical analyses were performed using GraphPad Prism 7.0 software. All results are shown as mean ± standard deviation (SD) of multiple independent experiments. Normality and lognormality test was used. If data did not meet the criteria for normal distribution, nonparametric test was performed. Unpaired Student’s *t*-tests were used for two-group comparisons. Two-way ANOVA followed by Fisher’s LSD test was applied to estimate how the mean of a quantitative variable changes according to the levels of two categorical variables. Wilcoxon test were used for the paired samples that are not normally distributed in mice experiment. *P-*values < 0.05 were considered statistically significant.

## Supplementary information

Supplemental figures

Supplemental table

Original western blots

Reproducibility Checklist

## Data Availability

The datasets generated during and/or analyzed during the current study are available from the corresponding author on reasonable request.
