# Supplementary material for: Discovery of an OTUD3 inhibitor for the treatment of non-small cell lung cancer
Source: Cell Death Dis. 2023 Jun 27;14(6):378. doi: 10.1038/s41419-023-05900-2 (PMC10300026; doi:10.1038/s41419-023-05900-2)
Supplement: Supplementary file 1 — Supplemental figures [file 41419_2023_5900_MOESM1_ESM.docx]

Supplementary Materials for

**Discovery of an OTUD3 inhibitor for the treatment of non-small cell lung cancer**

Yonghui Zhang^*^, Tongde Du^*^, Na Liu^*^, Juan Wang, Lingqiang Zhang, Chun-Ping Cui, Chaonan Li, Xin Zhang, Bo Wu, Jinhao Zhang, Wenli Jiang, Yubing Zhang, Yuting Zhang, Hongchang Li^#^ and Peiyu Li^#^

Correspondence to: lipeiyu6301@163.com/lhc_lihongchang@126.com


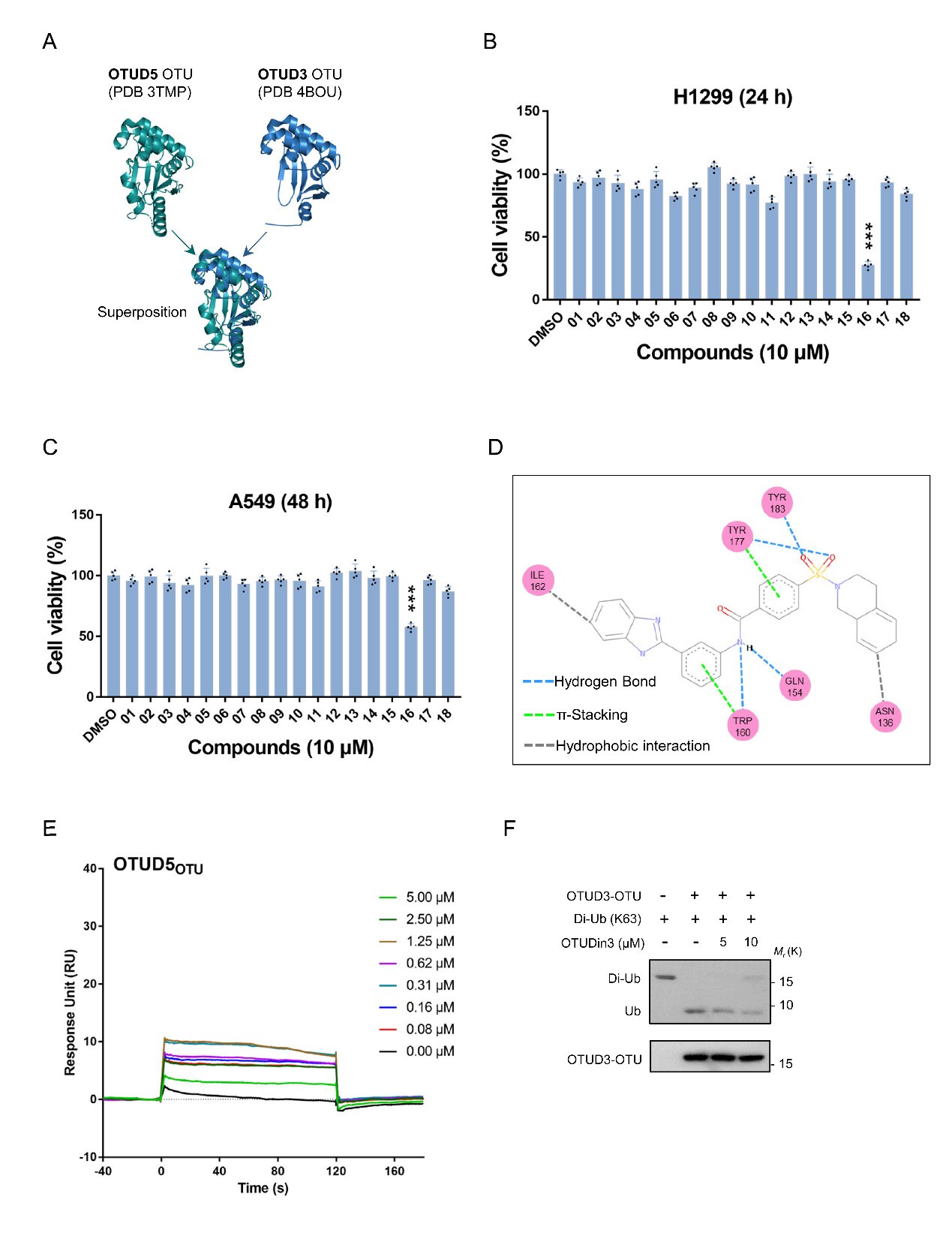


**Supplementary Figure 1. Identification of small-molecule OTUD3 inhibitor**

**A.** Superposition of OTU domain structures of OTUD3 and OTUD5. Structures are highly similar.

**B.** Cell proliferation assays showing that only compound 16 had a significant inhibitory effect on the proliferation of H1299 cell lines after 24 hours of treatment. Data shown are mean ± SD. n = 5 independent experiments. Student’s *t*-test. ****p* < 0.001, vs DMSO group.

**C.** Cell proliferation assays showing that only compound 16 had a significant inhibitory effect on the proliferation of A549 cell line after 48 hours of treatment. Data shown are mean ± SD. n = 5 independent experiments. Student’s *t*-test. ****p* < 0.001, vs DMSO group.

**D.** Schematic of the interactions between OTUDin3 and OTUD3. Compound interactions and surrounding residues are labeled.

**E.** Representative SPR sensorgram of OTUD5 OTU (aa 168-351) to measure affinity parameters of OTUDin3. OTUDin3 did not bind to OTUD5 .

**F.** OTUD3 OTU (aa 52-209) and K63-linked Di-Ubiquitin were incubated with indicated concentrations of OTUDin3 for 3 h at 37°C, followed by western blotting with indicated antibodies.

**E, F.** All panels are representative results of three independent experiments.


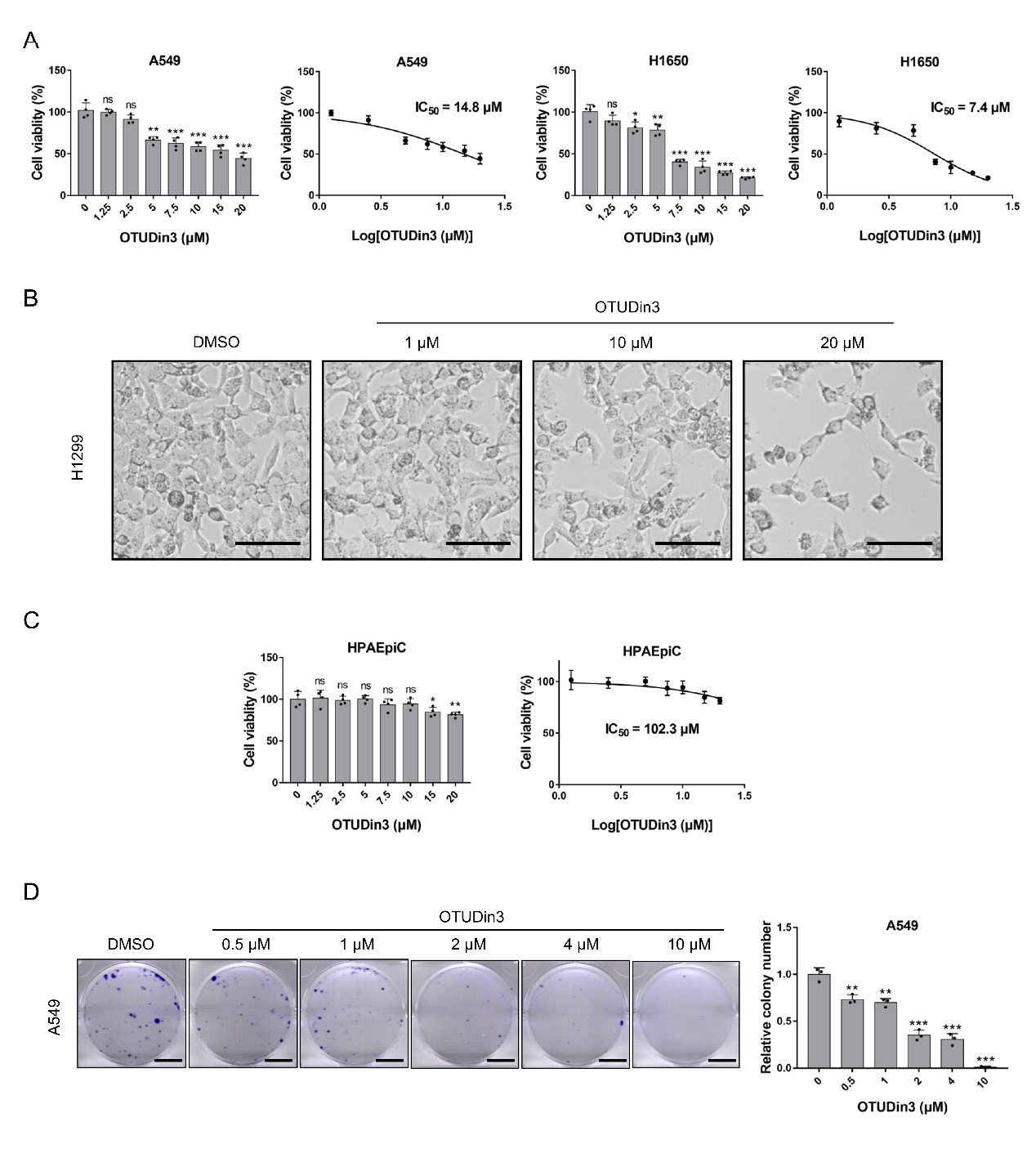


**Supplementary Figure 2. OTUDin3 inhibits lung cancer cell growth, migration and invasion, and induces apoptosis.**

**A.** Cell proliferation assays in A549 or H1650 cells treated with increasing concentrations of OTUDin3 for 72 h. Viability of cells was determined by the CCK-8 kit. IC_50_ was analyzed by nonlinear regression (curve fit) using GraphPad Prism V7.0 software.

**B.** H1299 cells were treated with indicated concentrations of OTUDin3 for 72h. Representative images photographed by using the optical microscope were shown. n = 3 independent experiments. Scale bar: 100 μm.

**C.** Cell proliferation assays in HPAEpiC cells treated with increasing concentrations of OTUDin3 for 72 h. Viability of cells was determined by the CCK-8 kit. IC_50_ was analyzed by nonlinear regression (curve fit) using GraphPad Prism V7.0 software.

**D.** Representative images of colony formation assays in A549 cells treated with increasing concentrations of OTUDin3 for two weeks after seeding of 1000 cells in six-well plate. Scale bar: 1 cm.

**A, C** and **D.** All the data shown are mean ± SD. n = 3 or 4 independent experiments. two-tailed unpaired Student’s *t*-test. **p* < 0.05, ***p* < 0.01, ****p* < 0.001, ns = no significance, vs control group.


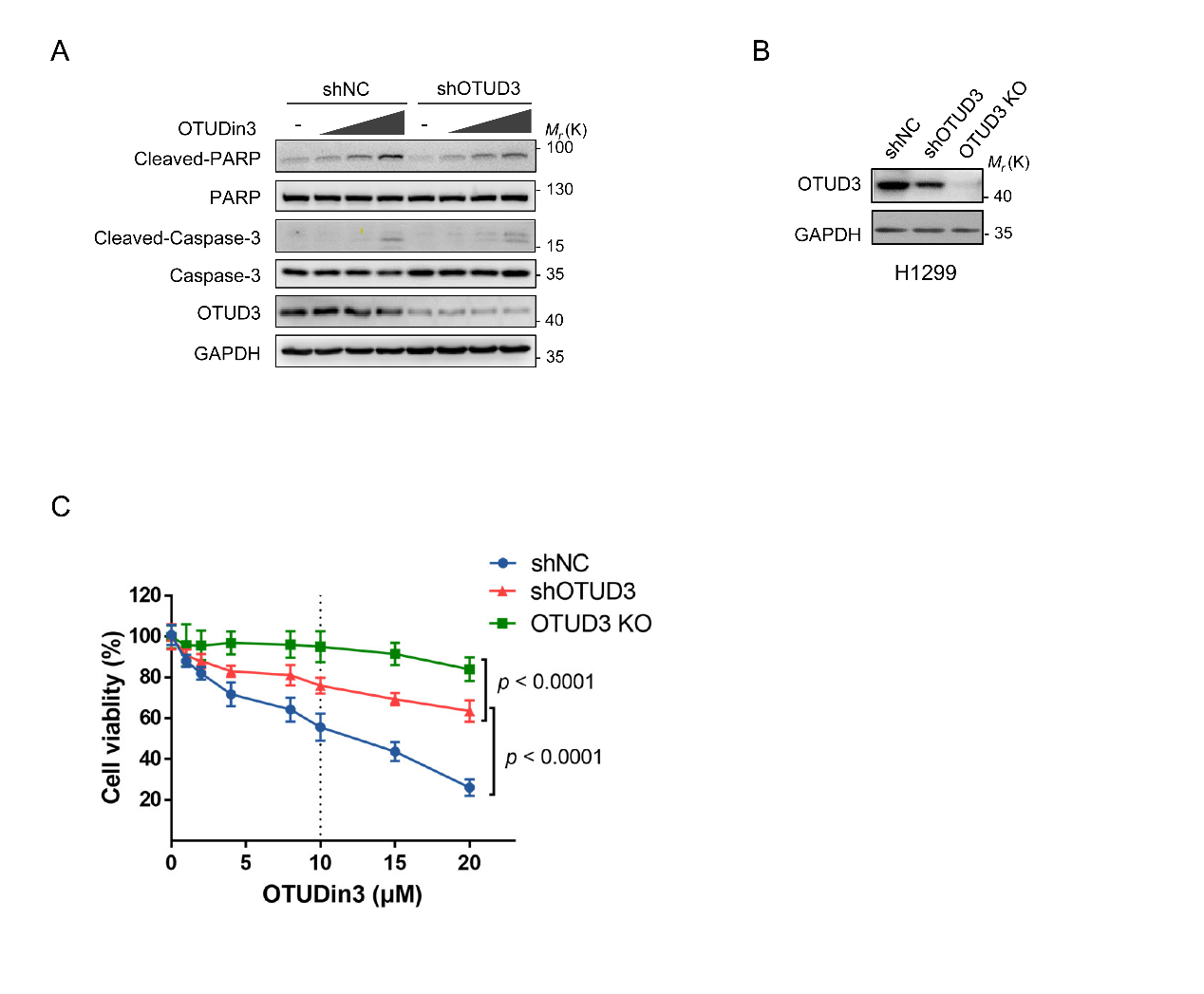


**Supplementary Figure 3.** **OTUDin3 inhibits NSCLC cell growth and induces apoptosis by targeting OTUD3.**

**A.** Western blot analyses of cell lysates were performed with antibodies against Cleaved-PARP, Cleaved-Caspase-3, PARP, Caspase-3, OTUD3, and GAPDH, as indicated. A representative image of three independent experiments is shown.

**B.** *OTUD3* was knocked out in H1299 cells using the CRISPR/Cas9 gene-editing technology. The protein levels of OTUD3 were analyzed by western blotting.

**C.** Cell proliferation assays in H1299 shNC, H1299 shOTUD3 and H1299 *OTUD3* KO cells treated with increasing concentrations of OTUDin3 for 72 h. Viability of cells was determined by the CCK-8 kit. All the data shown are mean ± SD. n = 5 independent experiments. Two-sided P values were calculated using two-way ANOVA. H1299 shNC vs H1299 shOTUD3, H1299 shOTUD3 vs H1299 *OTUD3* KO.


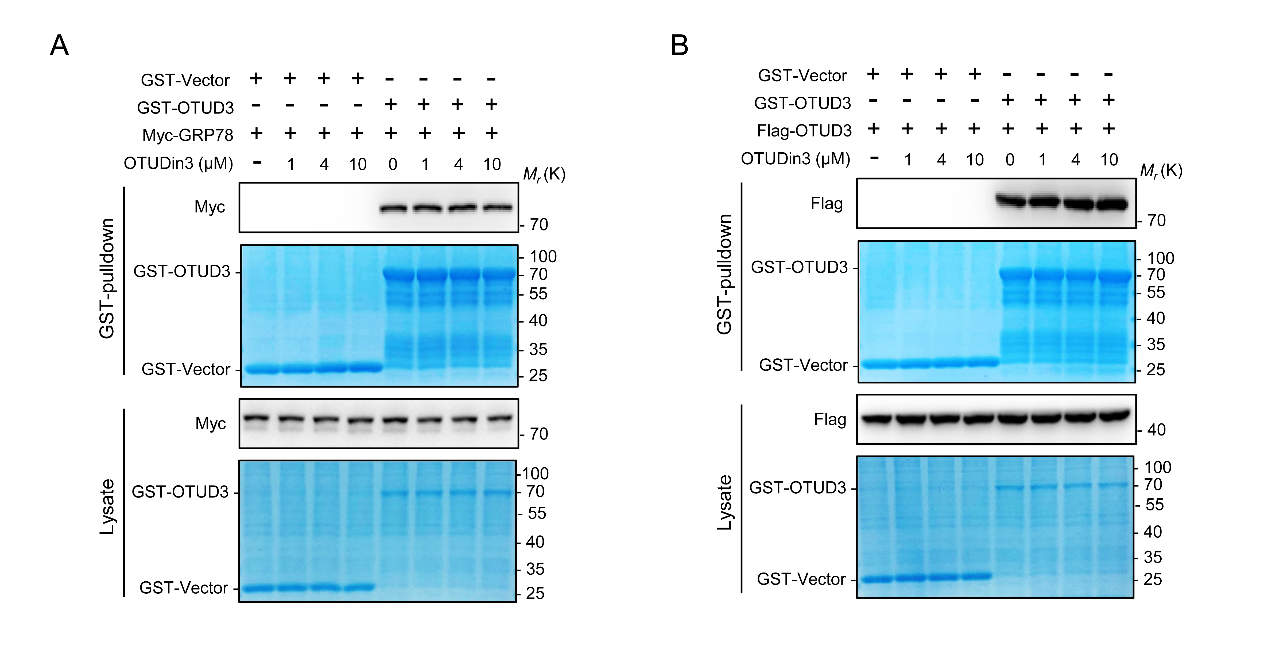


**Supplementary Figure 4. OTUDin3 does not interfere with the binding of OTUD3 to GRP78 and the dimerization of OTUD3.**

**A.** HEK293T cells transfected with Myc-GRP78 were lysed and lysates incubated with GST or GST-OTUD3 proteins with increasing concentrations of OTUDin3 treatment for 4 h. Proteins retained on Sepharose were blotted with the indicated antibodies, or visualized by SDS-PAGE and Coomassie blue staining.

**B.** Purified GST-OTUD3 and GST proteins were incubated with extracts from Flag-OTUD3 transfected HEK293T cells with increasing concentrations of OTUDin3 treatment for 4 h. Proteins retained on Sepharose were blotted with the indicated antibodies, or visualized by SDS-PAGE and Coomassie blue staining.

All panels are representative results of three or more independent experiments.
