# Supplementary material for: Discovery of an OTUD3 inhibitor for the treatment of non-small cell lung cancer
Source: Cell Death Dis. 2023 Jun 27;14(6):378. doi: 10.1038/s41419-023-05900-2 (PMC10300026; doi:10.1038/s41419-023-05900-2)

Fig. 1K

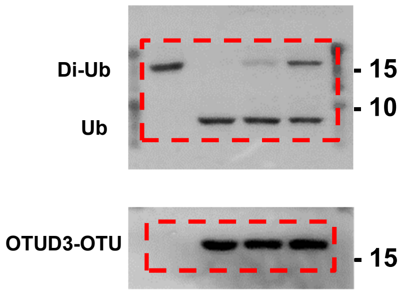

Fig. 1L

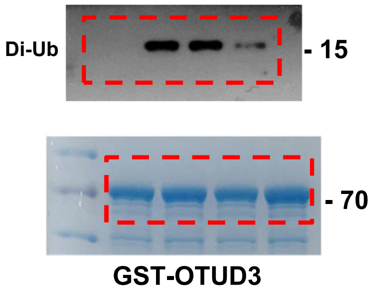

Fig. 2G

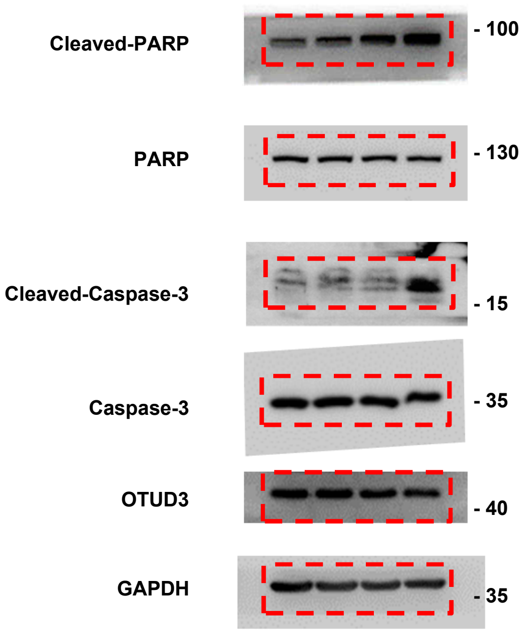
Cleaved-PARP
- 100
PARP
- 130
Cleaved-Caspase-3
- 15
Caspase-3
- 35
OTUD3
- 40
GAPDH
- 35

Fig. 3A

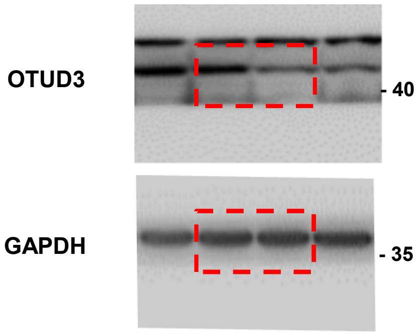

Fig. 4A

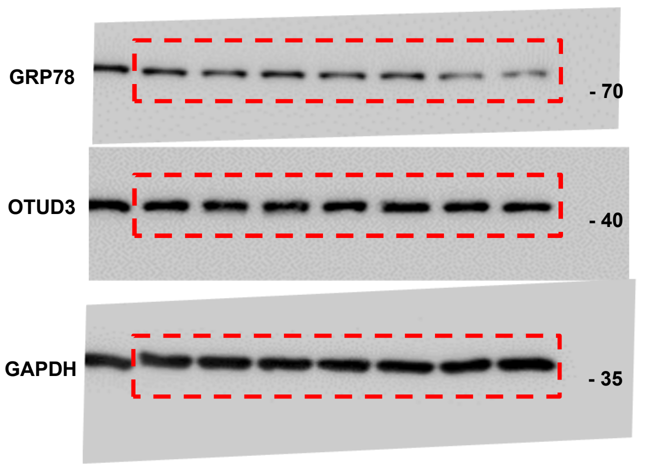

Fig. 4B

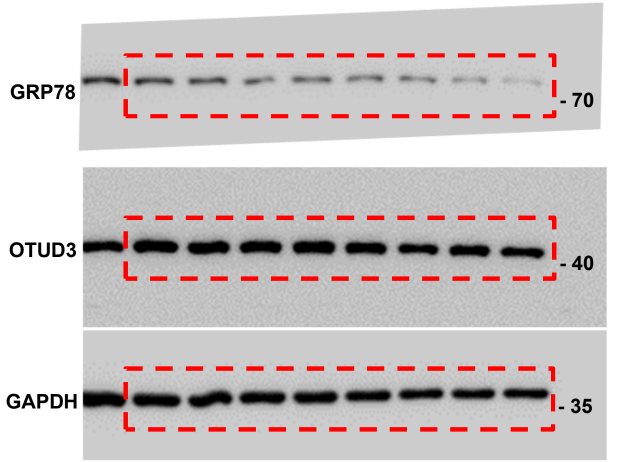

Fig. 4C

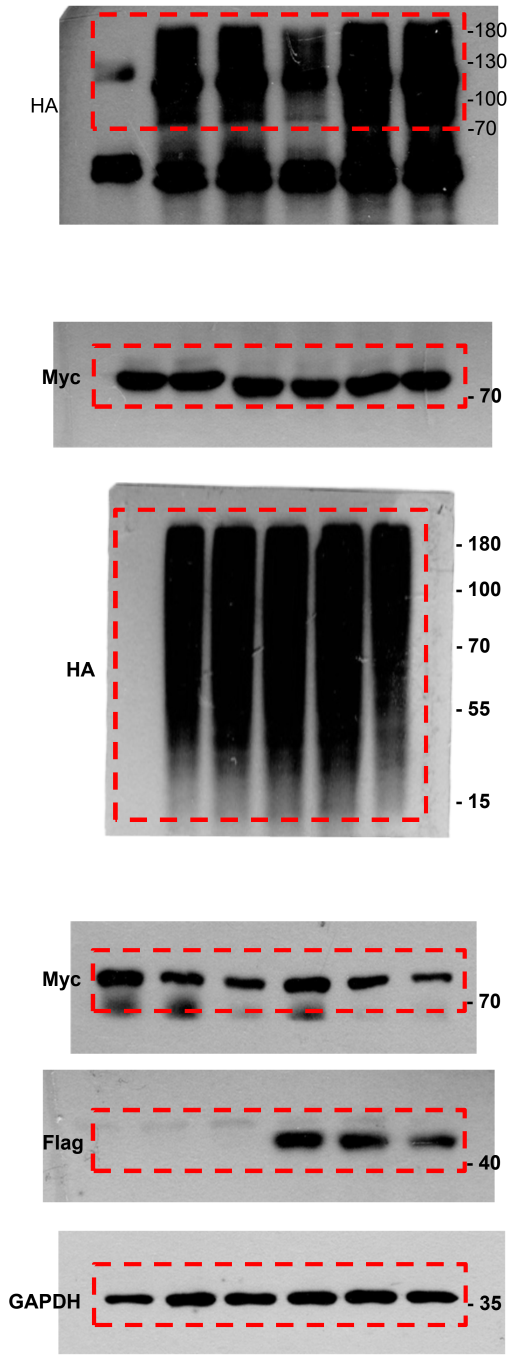

Fig. 4D

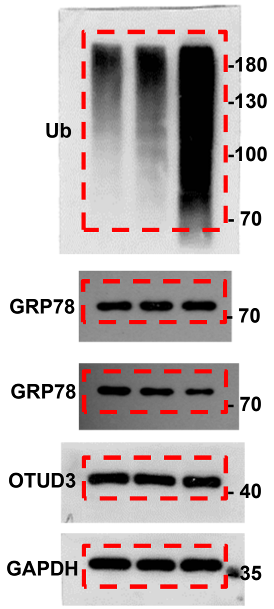

Fig. 4E

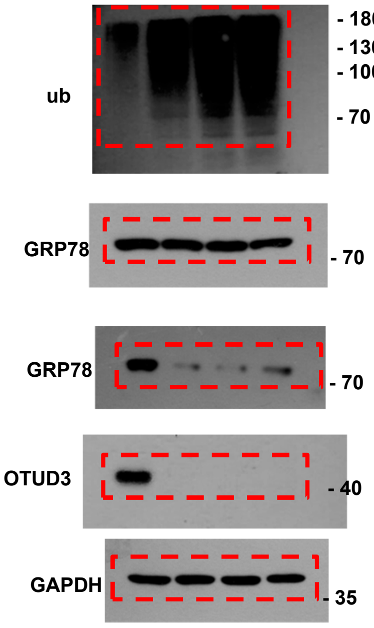

Fig. 4F

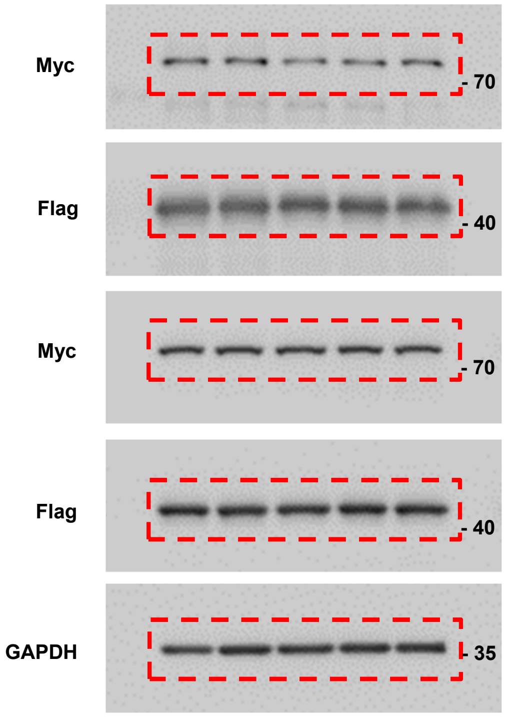

Myc
70
Flag
40
Myc
70
Flag
40
GAPDH
35

Fig. 4G

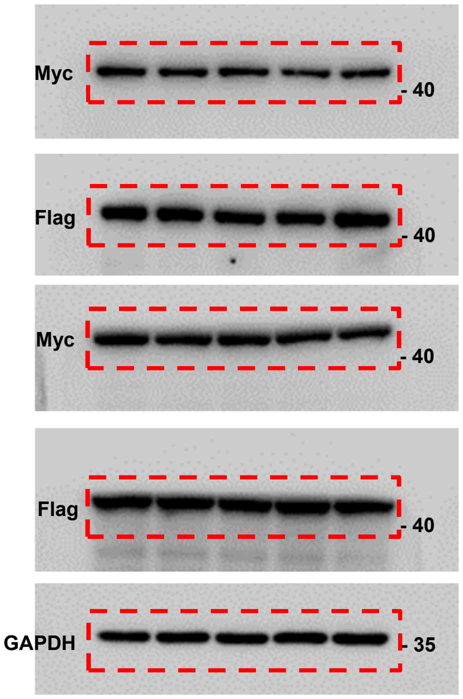

Myc
40
Flag
40
Myc
40
Flag
40
GAPDH
35

Supplementary Fig. 1F

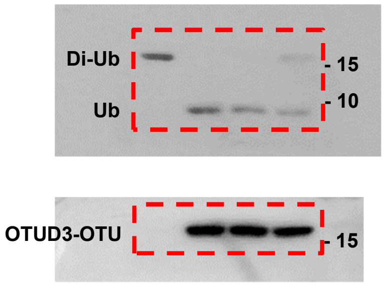

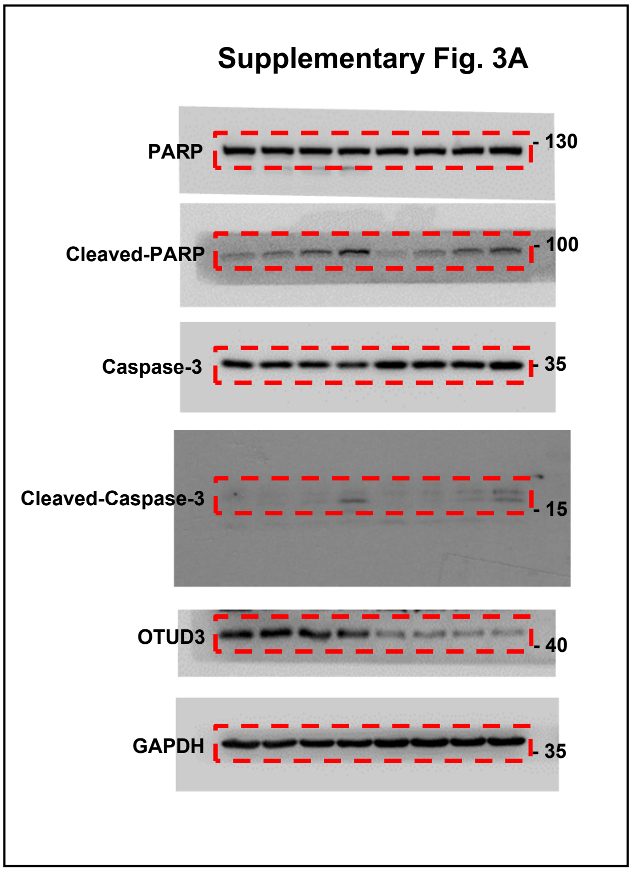
Supplementary Fig. 3A
PARP
- 130
Cleaved-PARP
- 100
Caspase-3
- 35
Cleaved-Caspase-3
- 15
OTUD3
- 40
GAPDH
- 35

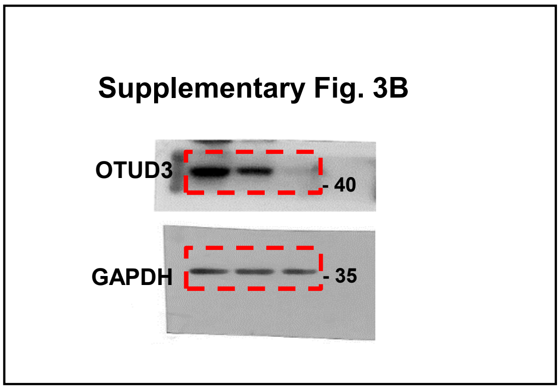
Supplementary Fig. 3B
OTUD3
- 40
GAPDH
- 35

Supplementary Fig. 4A

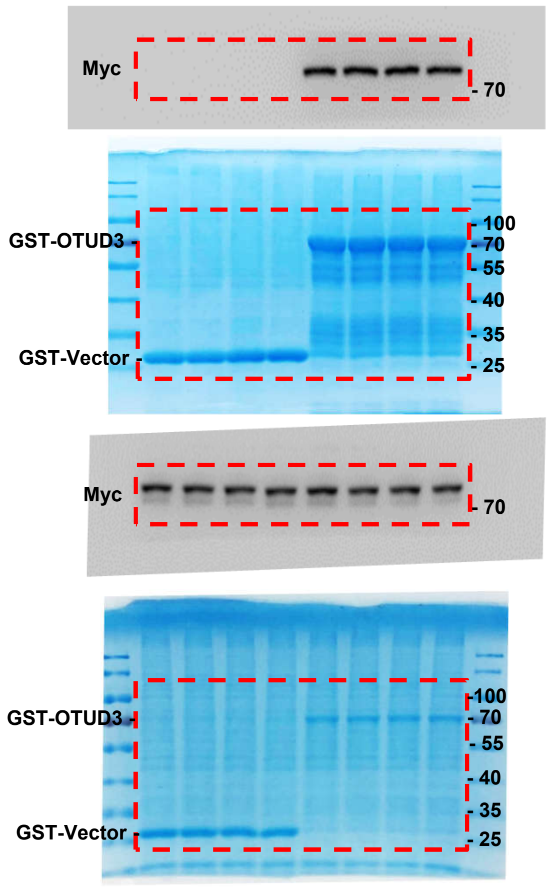

Supplementary Fig. 4B

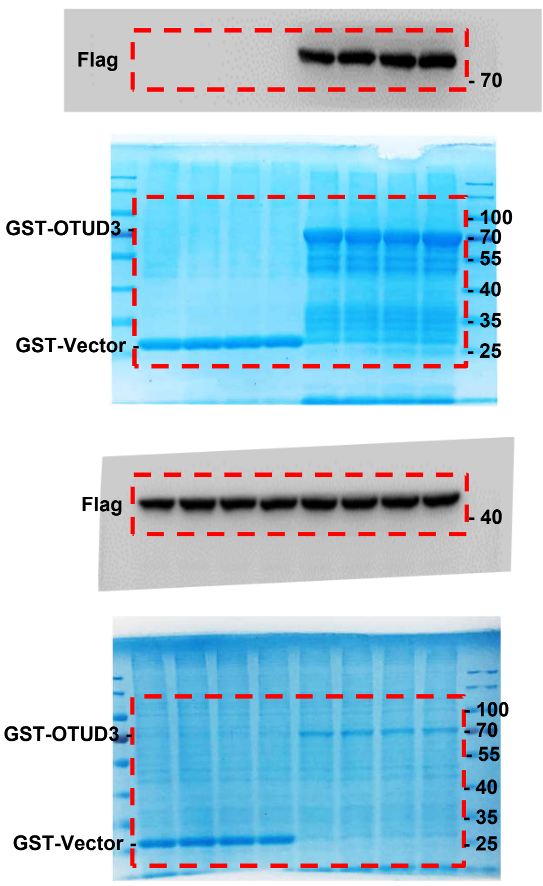

Supplement: Supplementary file 3 — Original western blots [file 41419_2023_5900_MOESM3_ESM.pdf]
